# RENEB interlaboratory comparison for biological dosimetry based on dicentric chromosome analysis and cobalt-60 exposures higher than 2.5 Gy

**DOI:** 10.1038/s41598-025-89966-2

**Published:** 2025-02-14

**Authors:** Martin Bucher, David Endesfelder, Stefan Pojtinger, Ans Baeyens, Joan-Francesc Barquinero, Christina Beinke, Laure Bobyk, Eric Gregoire, Rositsa Hristova, Juan S. Martinez, Prabodha Kumar Meher, Marcela Milanova, Octávia Monteiro Gil, Alegria Montoro, Jayne Moquet, Mercedes Moreno Domene, María Jesús Prieto, Monica Pujol-Canadell, Mingzhu Sun, Georgia I. Terzoudi, Ales Tichy, Sotiria Triantopoulou, Marco Valente, Anne Vral, Andrzej Wojcik, Ursula Oestreicher

**Affiliations:** 1 Department of Effects and Risks of Lonising and Non-Ionising Radiation, Federal Office for Radiation Protection (BfS), Oberschleissheim, Germany; 2 Department for Dosimetry for Radiation Therapy and Diagnostic Radiology, Physikalisch- Technische Bundesanstalt (PTB), Braunschweig, Germany; 3 Radiobiology Lab, Department of Human Structure and Repair, Ghent University, Gent, Belgium; 4 Unitat d’Antropologia Biològica, Departament de Biologia Animal, Biologia Vegetal i Ecologia, Universitat Autònoma de Barcelona, Bellaterra, E-08193 Catalonia Spain; 5 Bundeswehr Institute of Radiobiology, Munich, Germany; 6 Department of Radiation Biological Effects, French Armed Forces Biomedical Research Institute, Brétigny-sur-Orge, France; 7 Institut de Radioprotection et de Sureté Nucléaire (IRSN), PSE-SANTE / SERAMED / LRAcc, Fontenay-aux-Roses, F- 92260 France; 8 Radiobiology Department, National Centre of Radiobiology and Radiation Protection, Sofia, Bulgaria; 9 Center for Radiation Protection Research, Department of Molecular Biosciences, The Wenner- Gren Institute, Stockholm University, Stockholm, Sweden; 10 Department of Radiobiology, Military Faculty of Medicine, University of Defence, Hradec Kralove, Czech Republic; 11 Centro de Ciências e Tecnologias Nucleares, Departamento de Engenharia e Ciências Nucleares, Instituto Superior Técnico (IST), Universidade de Lisboa, Lisboa, Portugal; 12 Service of Radiological Protection, Clinical Area of Medical Image, University and Polytechnic La Fe Hospital, Valencia, Spain; 13 Radiation Effects Department, UK Health Security Agency, Radiation, Chemicals, Climate and Environmental Hazards Directorate, Chilton, UK; 14 Laboratorio de Dosimetría Biológica. Servicio de Oncología Radioterápica, Hospital General Universitario Gregorio Marañon, Madrid, Spain; 15 Health Physics, Radiobiology and Cytogenetics Laboratory, Institute of Nuclear and Radiological Sciences and Technology, Energy and Safety, National Centre for Scientific Research ‘Demokritos’, Athens, Greece

**Keywords:** Ionising radiation, Biological dosimetry, Dicentric chromosome, Interlaboratory comparison, Network, Radiation accident, Cytogenetics, Chromosomes, Diagnostic markers

## Abstract

In previous RENEB interlaboratory comparisons based on the manual scoring of dicentric chromosomes, a tendency for systematic overestimation for doses > 2.5 Gy was found. However, these exercises included only very few doses in the high dose range, and they were heterogeneous in terms of radiation quality and evaluation mode, and comparable only to a limited extent. Here, this presumed deviation was explored by investigating three doses > 2.5 Gy. Blood samples were irradiated (2.56, 3.41 and 4.54 Gy) using a ^60^Co source and sent to 14 member laboratories of the RENEB network, which performed the dicentric chromosome assay (manual and/or semi-automatic scoring) and reported dose estimates. Most participants provided estimates that agreed very well with the physical reference doses and all provided dose estimates were in the correct clinical category (> 2 Gy). The previously observed tendency for a systematic bias across all laboratories was not confirmed. However, tendencies for systematic underestimation were detected for dose estimations for reference doses given in terms of absorbed dose to blood and for some participants, a laboratory-specific trend of systematic under- or overestimation was observed. The importance of regularly performed quality checks for a broad dose range became obvious to avoid misinterpretation of results.

**Supplementary Information:**

The online version contains supplementary material available at 10.1038/s41598-025-89966-2.

## Introduction

The analysis of dicentric chromosomes is supposed to be the “gold standard method” for dose estimation in biological dosimetry^1^. This cytogenetic assay is internationally established, standardised^2,3^, and is the most commonly used method^1^. This is based on the specificity for ionising radiation, the low background frequency in healthy controls, the good reproducibility of dose-effect curves, and the fact that age and gender have no influence on the results^4–6^.

In the context of biological dosimetry, dose-effect curves (calibration curves) are the basis to perform dose estimation and are derived from the analysis of blood samples irradiated ex vivo under known conditions^7^. For dicentric chromosomes, dose-effect curves are often established with up to 5 Gy for low linear energy transfer radiation^4^. However, ex vivo irradiations are only an approximation of reality and they are artificially biased by the experimental irradiation set-up. For example, dosimetry and calibration of the beam as well as the irradiation of blood samples can be performed in air, water or tissue-equivalent phantoms. It is therefore possible that calibration of the beam and irradiation of blood samples are not performed in the same setting and that the reference dose may need to be converted subsequently. This is particularly the case if there is a large time interval between experiments or if blind samples are not irradiated in the same facility as the dose-effect curve.

Biological dosimetry laboratories should therefore have high quality standards that are regularly verified. Participation in interlaboratory comparisons (ILCs) ensures high quality sample preparation, analysis and dose estimation. In addition, ILCs simulating a real accident situation train the workflows of all participating laboratories and contribute to the collaboration and preparation for a major radiological / nuclear (RN) incident. This is very important because the large number of samples in major RN incidents quickly exceeds the capacity of individual laboratories, and the formation of networks among specialised laboratories is therefore essential to ensure adequate and comparable dose assessment in such situations^8^. In Europe, the RENEB (Running the European Network of Biological Dosimetry and Retrospective Physical Dosimetry) network was founded for this purpose and the organisation of ILCs is a central focus of the network’s activities^9,10^. Due to the participation of many different partner laboratories and the large amount of data collected, a major advantage of RENEB is that the ILCs enable the identification of problems, as for instance regarding the delivery of samples or the presence of systematic deviations in the methodological procedures of the participants.

In previous ILCs of the RENEB network based on the manual scoring of dicentric chromosomes, a tendency for systematic overestimation for doses above 2.5 Gy was found^11,12^. However, due to other scientific priorities only three out of 19 reference doses were higher than 2.5 Gy in the past ILCs and in all cases only a dose estimation based on a small number of cells, as it is common in the triage scoring mode, was performed^12^. The aim of this ILC was therefore the validation of the previously observed trend of a systematic overestimation of doses above 2.5 Gy.

## Results

For this RENEB ILC, blood samples were irradiated ex vivo in air with three doses (air kerma free in air 2.56; 3.41 and 4.54 Gy / absorbed dose to blood 2.71; 3.60 and 4.80 Gy) using a ^60^Co source. These doses were defined as the “reference doses” for this ILC. Blood samples were blinded and coded (reference dose 1, 2, 3) and sent to 14 member laboratories of the RENEB network, which performed the dicentric chromosome assay using their own protocol and reported point estimates of the dose with corresponding 95% confidence intervals.

### Dose-effect curves

**Fig. 1:**
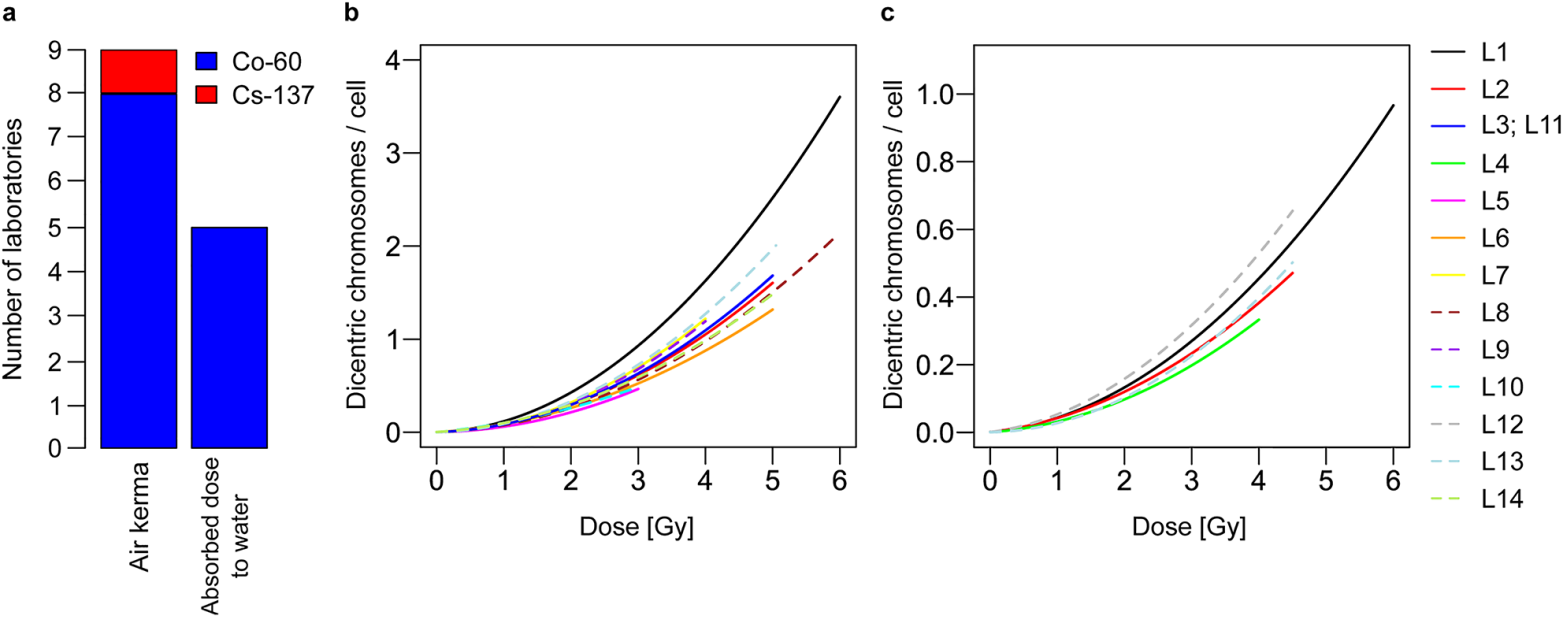
Dose-effect curves from participating laboratories. (**a**) Number of laboratories (y-axis) where the source used for the calibration curve was calibrated in terms of air kerma or absorbed dose to water. Colours indicate the radiation type used for the establishment of the calibration curves. **(b & c)** Manually and semi-automatically scored linear-quadratic dose-effect curves. The participants were labelled as L1-L14 and displayed by different colours and line types.

To perform dose estimation of blind samples all participants used gamma-ray curves (without additional filtration) and apart from L3 (curve from Barquinero et al.^13^ which was used as an example in the IAEA manual^4^) all participants used their own calibration curves (Table 1). Two participants (L5 and L10) had calibration curves with a maximum dose of 3 Gy and three participants (L4, L7 and L9) with a maximum of 4 Gy. Thus, the maximum dose of the calibration curves was lower than reference dose (ref. dose) 2 and/or 3. For nine participants the radiation source for the calibration curve was calibrated in terms of air kerma and for five participants in terms of absorbed dose to water (Fig. 1a). Heterogeneity was also observed in terms of the temperature during the irradiation and in terms of irradiation in air or in water.

**Table 1:** Details about the irradiation conditions for the establishment of calibration curves of each participant. The column “Curve” indicates whether a participant used a curve established in its own laboratory or a curve from another source. The columns “No. doses” and “Maximum dose” indicate the number of dose points and maximum dose used for calibration curves. For columns “Number doses”, “Maximum dose” and “Blood samples irradiated in”, parameters for manual and semi-automatic (in parentheses) scoring are provided and the hyphen “-” in the column indicates that the corresponding scoring mode was not performed or the information was not available. The column “Dosimetry” indicates whether the doses for the irradiations for the establishment of the calibration curve were given as air kerma or absorbed dose to water.

Code	Curve	Source	No. doses	Max. dose (Gy)	Dose rate (Gy/min)	Distance source to sample (cm)	Dosimetry	Medium irradiation	Temp. irradiation (°C)
L1	own	^137^Cs	12 (12)	6 (6)	0.446	15	air kerma	air (air)	20
L2	own	^60^Co	10 (11)	5 (4.5)	0.27	–	air kerma	water (-)	37
L3	IAEA*	^60^Co	11 (-)	5 (-)	1.07–1.175	81	water	water (-)	37
L4	own	^60^Co	− (11)	− (4)	0.5	67.8	air kerma	- (water)	37
L5	own	^60^Co	7 (-)	3 (-)	0.126–0.180	80	air kerma	air (-)	20
L6	own	^60^Co	11 (-)	5 (-)	0.5774	80	water	water	37
L7	own	^60^Co	8 (-)	4 (-)	0.745	200	air kerma	air (-)	20
L8	own	^60^Co	9 (-)	6 (-)	0.3	10	air kerma	air (-)	20
L9	own	^60^Co	9 (-)	4 (-)	0.2385	–	water	air (-)	20
L10	own	^60^Co	8 (-)	3 (-)	1	15	air kerma	air (-)	37
L11	own	^60^Co	11 (-)	5 (-)	1.07–1.175	81	water	water (-)	37
L12	own	^60^Co	− (12)	− (4,5)	0.166	–	water	- (water)	37
L13	own	^60^Co	15 (8)	5.05 (4.5)	0.27	–	air kerma	air (water)	37
L14	own	^60^Co	10 (-)	5 (-)	0.72	100	air kerma	air (-)	21

* curve from Barquinero et al.^13^ which was used as an example in the IAEA manual^4^.

The calibration curves were heterogeneous (Fig. 1b and c and Supplementary Table S1) with linear coefficients α (coefficient of dicentrics per cell per unit dose (units Gy^− 1^)) ranging between 0.01 and 0.069 (manual scoring) or 0.003–0.026 (semi-automatic scoring) and linear-quadratic coefficients β (coefficient of dicentrics per cell per unit dose squared (units Gy^− 2^)) ranging between 0.031 and 0.097 (manual scoring) or 0.003–0.026 (semi-automatic scoring).

### Dispersion of scoring results

First, the dispersion index (δ), which is defined by the variance (σ^2^) divided by the mean (µ) (Eq. 1), of all scoring results was evaluated.

1 $$\:\delta\:=\:\frac{{\sigma\:}^{2}}{\mu\:}$$

For manual scoring, a tendency for underdispersion (δ < 1) was observed for the blind sample with the highest dose (ref. dose 3) with a mean of δ = 0.92. For samples ref. dose 1 and 2, the dispersion indices were distributed around 1 and had a mean of 0.97 for both blind samples (Supplementary Figure S1). In contrast, for semi-automatic scoring, overdispersion (δ > 1) was observed for all blind samples with mean values of 1.06 (ref. dose 1), 1.07 (ref. dose 2), 1.11 (ref. dose 3) and the U-test was significant for four of five provided results for all three blind samples (Supplementary Figure S1).

### Dose estimation of blind samples

The participants were asked to provide point estimates of the dose with a, corresponding 95% confidence interval (CI). A total of 13 participants provided dose estimates. Due to problems with slide preparation L5 did not perform dose estimations and L14 did the analysis based on slides provided by L1. For dose and uncertainty assessment, 12 laboratories used the Biodose Tools software^14^ as recommended for this ILC, while one laboratory used the CABAS software^15^, which calculates the 95% CI without taking the error of the dose-effect curve into account. Manual scoring was performed by 11 and semi-automatic scoring by five participants. This means that eight participants performed only manual scoring, two only semi-automatic scoring (L4, L12) and three both scoring methods (L1, L2, L13). In total, 7,379 and 52,713 metaphases were analysed manually or semi-automatically, respectively.

**Fig. 2:**
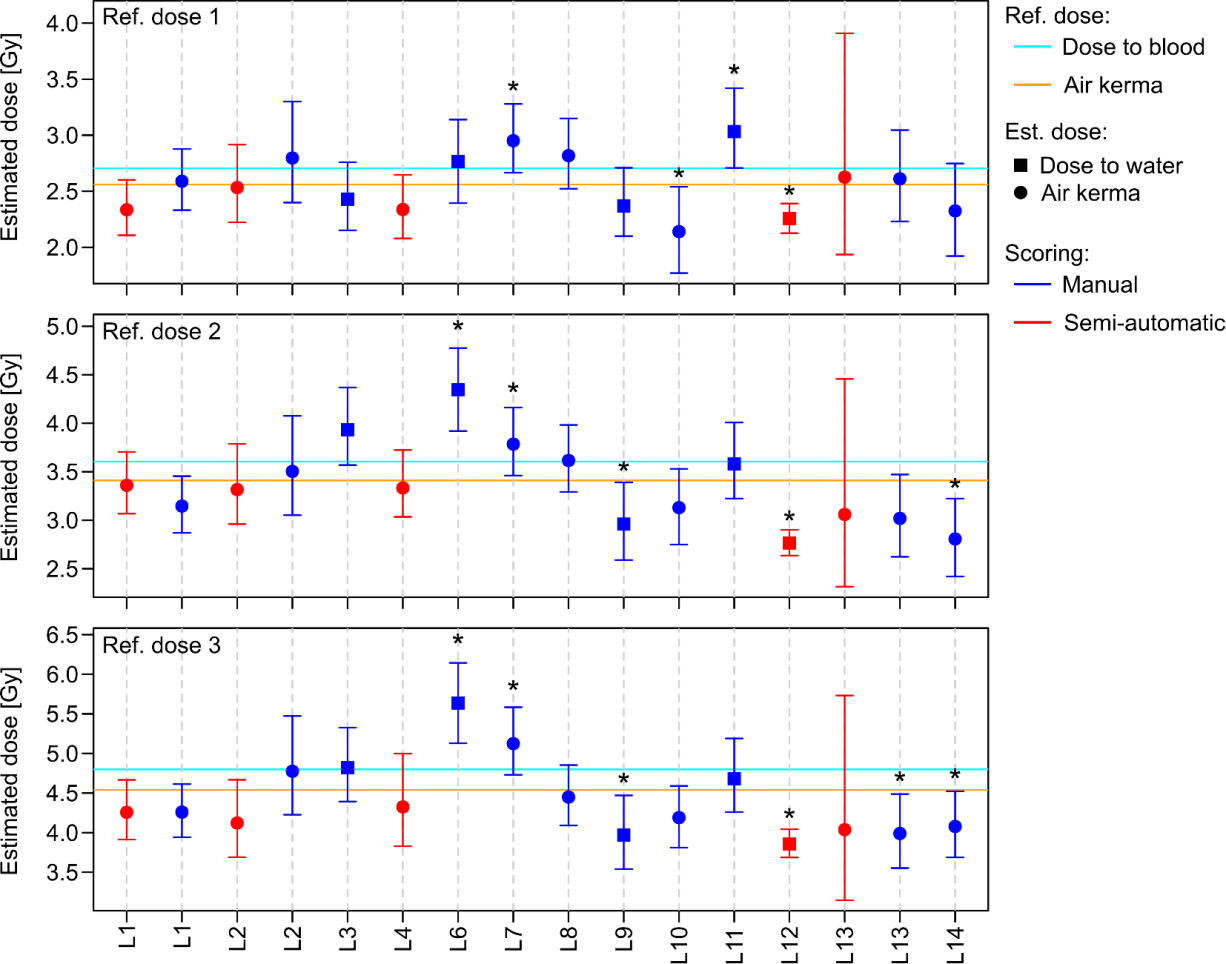
Dose estimates and 95% confidence intervals. The figure shows the point estimates of the dose and the corresponding 95% confidence intervals (error bars) for each blind sample / reference dose for each participating laboratory (L1-L14). Manually scored results are shown in blue and semi-automatically scored results in red. Reference doses in terms of air kerma or absorbed dose to blood are shown by orange and cyan horizontal lines, respectively. Results where the 95% confidence interval does not include the reference dose are indicated by asterisks *. The symbols indicate whether the source used for the establishment of the calibration curves used for dose estimation was calibrated in terms of air kerma (=) or dose to water (< ).

Compared to the reference dose given in terms of air kerma and absorbed dose to blood, for manual scoring 73% and 82% (ref. dose 1), 55% and 45%(ref. dose 2) or 55% and 45% (ref. dose 3) and for semi-automatic scoring 80% (for all blind samples and definitions of the reference doses) of the participants included the reference dose in the estimated 95% CI. When dose estimates of the blind samples, obtained with calibration curves established in terms of air kerma or absorbed dose to water, were compared with the corresponding definitions (air kerma or dose to blood) of the reference dose (matching reference dose), for manual scoring 73% (ref. dose 1), 64% (ref. dose 2) or 55% (ref. dose 3) and for semi-automatic scoring 80% (for all blind samples) of participants included the reference dose in the estimated 95% CIs (Fig. 2).

**Fig. 3:**
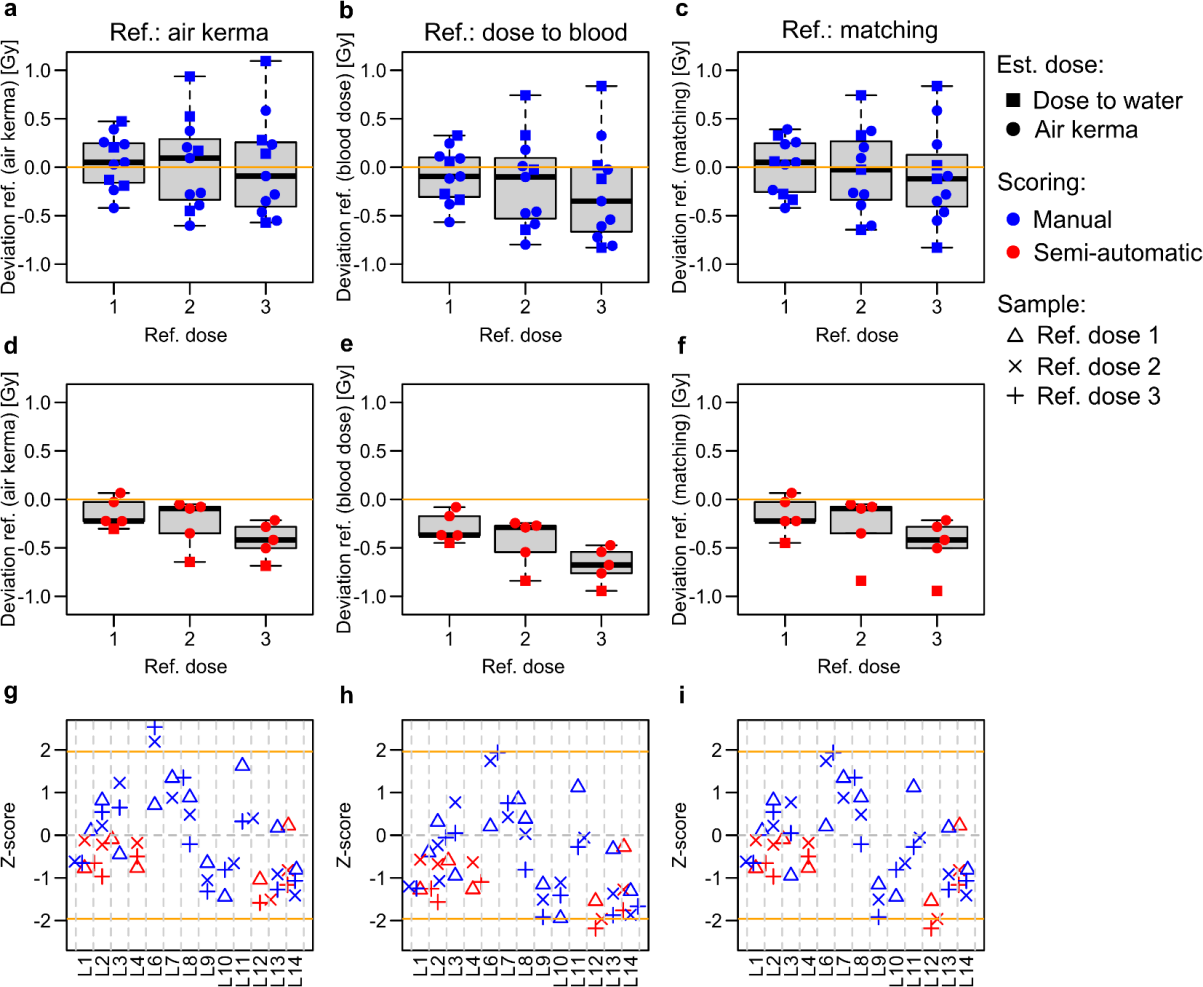
Deviations from reference doses and Z-scores. (**a**-**f**) Boxplot of the deviation (in Gy) of the dose estimates compared to reference doses (orange line) in terms of air kerma (**a & d**), absorbed dose to blood (**b & e**) or matching the dose definition of the applied calibration curve (**c & f**). Manually scored results are shown in blue (**a-c**) and semi-automatically scored results in red (**d-f**). The symbols indicate whether the source used for the establishment of the calibration curves used for dose estimation was calibrated in terms of air kerma (=) or dose to water (< ). **(g-i)** Z-Scores relative to reference doses in terms of air kerma (**g**) or dose to blood (**h**) or matching the dose definition of the applied calibration curve (**i**). The orange lines indicate the thresholds for questionable results (± 1.96). The symbols indicate the blind samples △ ref. dose 1; ref. dose 2; ref. dose 3).

While no general trend for systematic under- or overestimation could be observed for manual scoring compared to the reference dose in terms of air kerma or for matching reference doses (Fig. 3a and c), a tendency for underestimation could be observed for the highest dose compared to reference doses given in terms of dose to blood (Fig. 3b). For semi-automatic scoring, a general tendency for underestimation was observed with increasing dose, especially for reference doses given in terms of dose to blood (Fig. 3d-f).

In addition, some laboratory-specific systematic under- or overestimation was observed for manual scoring. The results of a participating laboratory were defined to show systematic under-/overestimation if (1) all three samples were consistently under-/overestimated and (2) if at least two of the 95% confidence intervals did not include the reference dose. For manual scoring, systematic overestimation was observed for L6 and L7 and systematic underestimation for L9 and L14 (Figs. 2 and 3g and i). For L6 and L7, a relatively low (L6) or intermediate (L7) number of dicentric counts would be expected from the calibration curve and an intermediate to high number of dicentric counts was observed (Supplementary Figure S2). In contrast, for L9 and L14, an intermediate number of dicentric counts was expected and a low number of dicentric counts was observed (Supplementary Figure S2). Interestingly, there was a significant (*P* < 0.05) correlation between the provided dose estimates of all three blind samples (Supplementary Figure S3). Especially between the two samples with the highest doses (ref. dose 2 and ref. dose 3), the correlation was very strong, suggesting that the same trend for too low or too high dose estimates of some laboratories was present for both samples (Supplementary Figure S3).

Although there were some laboratories with systematic under- or overestimation, the analysis of Z-scores revealed only one participant (L12) with two questionable Z-scores (Z > 2) for ref. doses 2 and 3 compared to the matching reference dose definition (Fig. 3i).

## Discussion

**Fig. 4:**
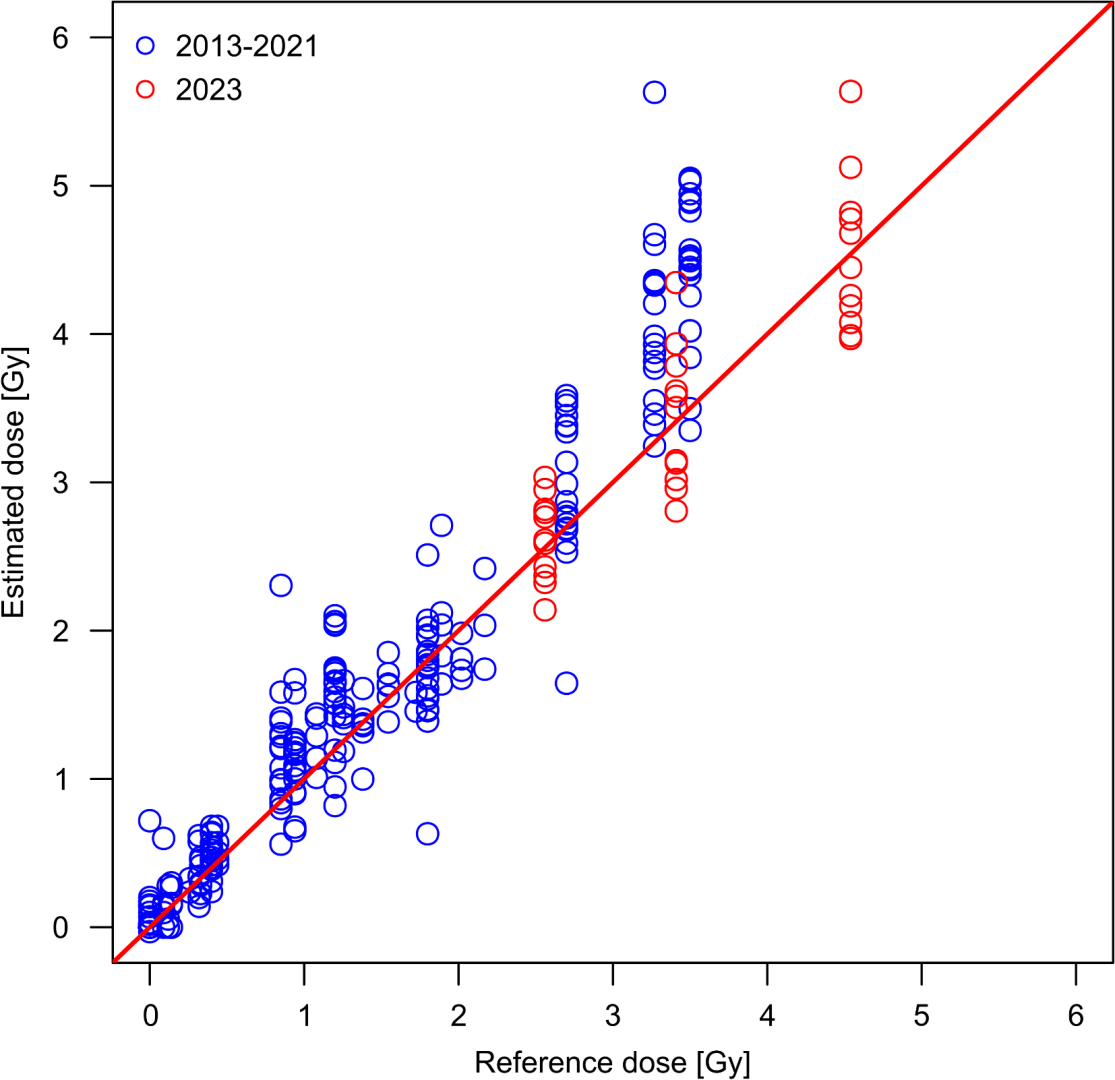
Comparison of dose estimates to RENEB ILCs in the past. The reference doses (x-axis) and the corresponding dose estimates (y-axis) from RENEB member laboratories are shown for all ILCs conducted from 2013–2023. Only samples simulating homogeneous whole-body exposures and manual scoring are shown for each exercise. The orange solid line shows the bisecting line. The results of past ILCs are shown in blue and the results of the current ILC in red.

In the event of a major RN incident, there is a possibility that a large number of the affected individuals will be exposed to high doses > 2.5 Gy^16^. In such situations, there is a high need for information for the optimal medical management of affected individuals^17^. Biological dosimetry can make a valuable contribution to the identification and classification of exposed individuals, the verification of the exposure to high doses and the support of medical management^18^. However, specialised laboratories need to form operational networks to handle the potentially large number of samples and regularly train their collaborations within ILCs to assist in these situations^8,9,19–21^. The RENEB network currently brings together 16 institutions (voting members) and 43 researchers (associate members) across Europe/ Asia and organises an ILC every two years to ensure high quality dose assessment and to improve the performance of its members. A comparison of dose estimates based on the manual scoring of dicentric chromosomes for six previous RENEB ILCs (2013–2021) showed a tendency for overestimation for higher doses (i.e., above 2.5 Gy; Fig. 4)^12^. There was some variability regarding the definition of the reference doses in terms of air kerma or absorbed dose to water and also the type of radiation source used for these ILCs (Table 2).

**Table 2:** Information about sources, dosimetry and reference doses for recent RENEB ILCs.

Year	Dosimetry	Source	Blind doses (Gy)*
2013	air kerma	^137^Cs	0; 0.94; 3.27
2014	air kerma	^137^Cs	0.85; 2.7
2015^**^	dose to water	^60^Co	0; 0.44; 1.08; 1.89
2017	dose to water	X-ray (4 MV)	0; 0.4; 1.8
2019^**^	dose to water	1.36 TBq ^192^Ir	0.05–2.17
2021	dose to water	X-ray (240 kVp, ~ 75 keV)	0; 1.2; 3.5
2023	air kerma dose to blood	^60^Co	2.56; 3.41; 4.54 2.71; 3.60; 4.80

* Only blind doses for homogeneous exposures are shown.

** Exercise jointly organized with EURADOS WG10.

Following the recommendation of Endesfelder et al.^12^, an ILC was performed in this study with special focus on doses > 2.5 Gy. Blood samples were irradiated under standardized conditions in air with three reference doses (air kerma free in air 2.56; 3.41 and 4.54 Gy) using a ^60^Co source. In addition, absorbed dose to blood was calculated by Monte Carlo simulation (2.71; 3.60 and 4.80 Gy). The blood samples were sent to 14 laboratories of the RENEB network (voting members), which performed the dicentric chromosome assay and reported point estimates of the dose with a corresponding 95% CI.

In total, 12 out of 14 laboratories participated as planned in this ILC and one laboratory analysed slides from another laboratory. As observed in previous RENEB ILCs^10,11,22,23^ the calibration curves of all laboratories were relatively heterogeneous. However, all provided dose estimates were > 2 Gy and therefore in the correct clinical category as defined during the MULTIBIODOSE project^24^. Moreover, almost all dose estimates were within the range of +/- 1 Gy of the reference dose^25^ and all laboratories were able to determine the correct ranking of blind samples with increasing doses.

In contrast to the tendency of systematic overestimation of reference doses > 2.5 Gy in previous RENEB ILCs^12^, no systematic deviation was observed for manual scoring in this study and it can be assumed that no general bias is present for high doses (Fig. 4). Compared to previous RENEB ILCs, the present study systematically analysed high reference doses with the same radiation quality and a high number of cells per reference dose and laboratory. Previous interlaboratory comparisons investigated different research questions and were therefore heterogeneous in terms of radiation quality, dose definition and evaluation mode, and comparable only to a limited extent^10–12,22,23,26^. However, this heterogeneity can also be an advantage, as it allowed the in-depth analysis of aspects that have arisen during the different ILCs.

The results of semi-automatic scoring indicated a heterogeneous or partial body exposure (overdispersion) even though the exposure was homogeneous (Supplementary Figure S1). This study showed that if semi-automatic scoring were directly compared to manual scoring, a tendency for underestimation was observed with increasing dose. However, calibration curves for the two scoring types are innately different in terms of metaphase selection and aberration detection. Moreover, quality of the metaphases has a higher impact for semi-automatic than for manual scoring. A possible reason for the observation of underestimation might be differences in the quality of metaphases between the calibration curve and the blind samples. In addition, the significance of this observation is limited by the fact that only five laboratories carried out semi-automatic scoring and one of these five laboratories seemed to have a general bias underestimating all three blind samples. Thus, further research is required to understand the behaviour of semi-automatic scoring for high doses.

In addition, a tendency for underestimation with increasing dose was observed when comparing the results to the reference dose in terms of absorbed dose to blood for both scoring modes. This is very likely attributed to the fact that most laboratories have established their calibration curves in terms of air kerma and provided dose estimates in air kerma. This effect was reduced when the dose estimates and the reference dose had the same (matching) definition of the dose (both air kerma or absorbed dose to blood/water). In a real accident situation, the estimation should always be performed in terms of dose to blood or dose to water to avoid systematic bias^7^. The results of this study indicate that laboratories should be aware of the influence of differences in dose definitions between the dose-effect curve and sample used for dose estimation and should be able to provide dose estimates in terms of dose to blood or dose to water in future.

Furthermore, there were a few participants where the provided dose estimates suggested a laboratory-specific systematic under- or overestimation (consistent under- or overestimation for all dose estimates for the three reference doses and at least two of the 95% confidence intervals did not include the reference dose) for the three blind samples regardless of the reference dose definition (air kerma or dose to blood). For L6 and L7 a systematic overestimation and for L9 and L14 a systematic underestimation was observed. Two of these laboratories estimated doses for ref. dose 3 that were outside the range of their calibration curves (Table 1). This extrapolation is generally not recommended^2^ and could be one reason for deviations. Two laboratories reported that their calibration curve had been established some years ago and that there had been a change in laboratory staff. One laboratory only evaluated slides prepared from another laboratory and performed dose estimation afterwards with their own calibration curve. The preparation and quality of the slides could affect the scoring and lead to incomparability with the laboratory specific scoring procedure and a systematic deviation. This result is consistent with a previous study which showed that dose estimation is satisfactory when analysing image files from other laboratories and using one’s own dose-effect curve, but that there may be individual variations^26^.

The laboratory-specific under-/overestimation might suggest that the applied calibration curves of these participants do not anymore correspond to the scoring for the blind samples of this ILC. The reasons for this can be very different and range from the age of the calibration curve, improvement in technical equipment (microscope, camera or software) and changes in the laboratory staff over time. Therefore, according to ISO 19,238 ^2^ and the IAEA manual^4^, it is strongly recommended that biological dosimetry laboratories regularly perform internal performance checks of sample protocol, scoring, dose estimation, reporting of results and interlaboratory comparisons for scorers^2^. In addition, participation in interlaboratory comparisons provides an objective evaluation of a laboratory’s performance and allows the identification of problems^2^.

In summary, biological dosimetry laboratories should have high quality standards that are regularly verified and require in-depth knowledge of beam characteristics, beam calibration, dosimetry, experimental settings, temperature during irradiation and surrounding material for their dose-effect curves. However, radionuclide sources are becoming increasingly rare due to high maintenance costs and radiation protection issues, so that in some cases it is not possible to perform irradiations with quality-assured dosimetry close to the laboratory. In addition, the establishment of dose-effect curves for different radiation qualities is necessary, but labour-intensive, time-consuming and expensive when using a commercial irradiation facility. These requirements and developments represent an increasing challenge for many biological dosimetry laboratories in the future.

The collaboration in networks could help to overcome some of these challenges and should provide assistance to all members when needed. ILCs are one aspect of mutual assistance and allow the identification of problems at different levels. In this ILC, a presumed systematic overestimation for reference doses above 2.5 Gy was not confirmed. On the contrary, this ILC provides guidance for the organisation of future ILCs and helps laboratories to improve their dose estimation. Therefore, for future ILCs it is recommended that the reference dose and the dose estimate of blind samples from all laboratories should be reported for the same dose definition, ideally in terms of absorbed dose to blood or dose to water. This is strongly recommended for dose estimation not only in ILCs but also in real accident situations and will improve the comparability of results from different laboratories, simplify and standardise the reporting of such dose estimates, and bring ILCs closer to reality.

Nevertheless, in the context of a real accident situation, the results of this RENEB ILC showed that the dose estimates from all participating RENEB laboratories could help to prioritise exposed individuals, assist medical management and provide a satisfactory dose estimate in almost all cases.

## Materials and methods

### Blood collection

Blood samples of a female healthy adult donor were obtained in heparinized 10 mL tubes (Sarstedt AG & Co. KG, Germany) by venepuncture. Blood collection and all methods were carried out in accordance with relevant guidelines and regulations to § 15 of the code of medical ethics for physicians in Bavaria and the ethics committee of the Bavarian Medical Association, Germany. Accordingly, there is no duty to advise and no ethical approval is required for quality assurance with anonymised data. Therefore, the approval for the study was waived along with the Bavarian Medical Association. Nevertheless, the study followed the principles of the Declaration of Helsinki. Blood collection was performed with signed informed consent by physicians.

### Irradiation setup

Blood samples were irradiated ex vivo in the blood collection tubes with a ^60^Co source (Research Institute of Atomic Reactors, Russia, GK60T03, Quelle 3) at the Physikalisch-Technische Bundesanstalt (PTB) in Braunschweig, Germany. The irradiation conditions for the ^60^Co source correspond to the “reference gamma radiation S-Co” from the ISO 4037-1 standard^27^. Irradiations were carried out in air with three doses (air kerma free in air, 2.56; 3.41 and 4.54 Gy) at an air kerma rate of 31.64 Gy / h, a source to surface distance of 43.76 cm, at room temperature (21 °C), at 1011 hPa and without any filters. These doses were defined as the ‘reference doses’ for this ILC. An acute, homogeneous whole-body γ-radiation exposure was simulated. To ensure homogeneous irradiation within the circular radiation field with a diameter of 14.7 cm, two irradiations were performed for each reference dose using two blood collection tubes each (four blood collection tubes in total). The two tubes were placed in a sample holder (made of polymethyl methacrylate (PMMA)) and irradiated vertically, with a distance of 4.3 cm between the tubes. The tubes were filled with 8.5 mL of blood, had a height of 8.4 cm and a diameter of 1.7 cm and were made of polypropylene with a high-density polyethylene (HDPE) cap, which was 2.5 cm high.

The air kerma rate free in air of the ^60^Co source was characterized with the PTB primary air kerma standard for ^60^Co gamma radiation^28^. For the irradiation of the blood collection tubes in this ILC, the air kerma rate was corrected to the time of irradiation based on the exponential decay of the ^60^Co source. The applied doses correspond to an air kerma free in air of 2.56; 3.41 and 4.54 Gy.

In addition, the absorbed dose to the blood inside the blood collection tubes was calculated based on Monte Carlo simulations using the Monte Carlo Framework EGSnrc^29^. Detailed models of the blood collection tubes were created for this purpose. Two simulations were carried out. One simulation was performed using the EGSnrc user code egs_kerma to simulate the air kerma at the reference position. A second simulation was performed using the EGSnrc user code cavity. Here, the models of the blood collection tubes were included into the simulation geometry in the same position as for irradiation. The blood dose was then calculated and compared with the results of the first simulation. Based on these simulations, the dose to blood for the described experiments was calculated (2.71, 3.60 and 4.80 Gy).

After irradiation the blood samples were incubated for 2 h at 37 °C to enable DNA damage repair^2^.

### Participating laboratories and shipment of blood samples

In total, 14 laboratories (voting members) from the RENEB network participated in this ILC. The laboratories were anonymized and named L1-L14.

Each laboratory received 2.1 mL of whole blood per sample, in total three samples. The reference doses were blinded and coded for all participating laboratories (reference dose 1, 2, 3). The blood samples for all laboratories outside Germany were shipped on the same day by express service according to standard regulations under UN 3373 Biological Substance Category B and in ambient temperature (temperature monitored). The blood samples for the two laboratories in Germany were transported by car on the same day. Blood samples reached 11 out of 14 laboratories within 24 h, two laboratories after 48 h and one laboratory after seven days. The temperature during transport ranged from 5 °C to 27 °C and was in the acceptable temperature range according to ISO 19,238 ^2^. L14 received slides prepared by L1.

### Dicentric chromosome assay

All laboratories had advanced experience in the analysis of dicentric chromosomes and dose estimation and followed all mandatory laboratory health and safety procedures in the course of conducting any experimental work reported here.

Each laboratory cultured lymphocytes for the dicentric chromosome assay according to its own protocols and following the recommendations of the IAEA^4^ and ISO standard 19,238 ^2^. In brief, lymphocytes in whole blood were cultured in cell culture medium and cell division was stimulated with phytohameagglutinin (PHA). Cells were blocked in metaphase stage of mitoses by addition of colcemid, and cell-cycle controlled scoring was ensured by addition of colcemid 24 h after starting the culture or using the Fluorescence plus Giemsa (FpG) staining method adding colcemid 45 h after starting to the cell suspension. After 48–52 h of culture, the cell suspension was processed by hypotonic treatment, fixation and several washing steps. The cell suspension was then transferred onto slides and stained accordingly.

All participants prepared the slides using their own standard staining method and performed a microscopic manual and/or semi-automated analysis to determine the number of dicentric chromosomes for dose estimation. For manual scoring, each participant was asked to score 200 cells per dose point. For the semi-automated scoring, each participant was asked to analyse as many cells as possible according to the laboratory’s own procedure in a real radiation accident situation.

All participants submitted an evaluation template including dose estimates, scoring results (including the number of cells analysed and the number and distribution of dicentric chromosomes), detailed information on culture variables, scoring variables and the calibration curve used.

### Dose estimation

All calibration curves were generated by fitting the yield of aberrations to linear-quadratic dose dependencies and the participants were asked to use their own calibration curves for dose assessment. Information on the details concerning the dose-effect curves (source, radiation quality, dose rate, distance, origin of curve, calibration of the radiation source based on air kerma or absorbed dose to water, irradiation temperature, irradiation in air or water, coefficients, number of analysed cells, number and distribution of dicentrics for the applied doses) was requested from the participants (see Table 1 and Table S1 for summary). For dose estimation as well as for the corresponding uncertainties it was recommended to use the Biodose Tools software^14^. Nevertheless, one laboratory performed dose estimations with the CABAS software, which does not account for the error of the curve^15^. The dose estimates and the corresponding 95% confidence interval (CI) were to be provided in Gy and according to the number of dicentric chromosomes scored.

### Statistical evaluation

In a first step, all provided dose estimates were quality checked by recalculating the dose estimates based on the provided calibration curve coefficients and the dicentric distribution of the test samples. The Z-Scores were calculated as described in Di Giorgio et al.^30^. To test whether the observed overdispersion is significantly different from 1, the U-test was applied as described in the IAEA manual and ISO 21243:2022 ^3,4^ and results with U > 1.96 were assumed to be significantly overdispersed (*P* < 0.05).

## Electronic supplementary material

Below is the link to the electronic supplementary material.

Supplementary Material 1

## Data Availability

The raw data supporting the conclusions of this article will be made available by the corresponding author, without undue reservation. Please contact the corresponding author of this article.
