# Supplementary material for: RENEB interlaboratory comparison for biological dosimetry based on dicentric chromosome analysis and cobalt-60 exposures higher than 2.5 Gy
Source: Sci Rep. 2025 Feb 14;15:5485. doi: 10.1038/s41598-025-89966-2 (PMC11828874; doi:10.1038/s41598-025-89966-2)
Supplement: Supplementary file 1 — Supplementary Material 1 [file 41598_2025_89966_MOESM1_ESM.docx]

Supplementary tables and figures

RENEB interlaboratory comparison for biological dosimetry based on dicentric chromosome analysis and cobalt-60 exposures higher than 2.5 Gy

Martin Bucher^1^*, David Endesfelder^1^, Stefan Pojtinger^2^, Ans Baeyens^3^, Joan F. Barquinero^4^, Christina Beinke^5^, Laure Bobyk^6^, Eric Gregoire^7^, Rositsa Hristova^8^, Juan S. Martinez^7^, Prabodha Kumar Meher^9^, Marcela Milanova^10^, Octávia Monteiro Gil^11^, Alegria Montoro^12^, Jayne Moquet^13^, Mercedes Moreno Domene^14^, María Jesús Prieto^14^, Monica Pujol-Canadell^4^, Mingzhu Sun^13^, Georgia I. Terzoudi^15^, Ales Tichy^10^, Sotiria Triantopoulou^15^, Marco Valente^6^, Anne Vral^3^, Andrzej Wojcik^8^ and Ursula Oestreicher^1^

**Supplementary Table S1: Linear-quadratic calibration curve coefficients (λ=C + αD + βD^2^) and corresponding standard errors (SE) for each participant (L1-L14) and scoring mode.** α is the coefficient of dicentrics per cell per unit dose (units Gy^−1^) and β is the coefficient of dicentrics per cell per unit dose squared (units Gy^−2^).

| **Code** | **Scoring mode** | **Calibration curve coefficients** | | | | | |
| --- | --- | --- | --- | --- | --- | --- | --- |
|  |  | **C** | **α** | **β** | **SE(C)** | **SE(α)** | **SE(β)** |
| L1 | semi-automatic | 0.0012 | 0.0184 | 0.0238 | 0.0003 | 0.0033 | 0.0014 |
|  | manual | 0.0012 | 0.0190 | 0.0969 | 0.0001 | 0.0041 | 0.0035 |
| L2 | semi-automatic | 0.0005 | 0.0240 | 0.0179 | 0.0002 | 0.0037 | 0.0015 |
|  | manual | 0.0008 | 0.0302 | 0.0581 | 0.0004 | 0.0057 | 0.0046 |
| L3 | manual | 0.0013 | 0.0210 | 0.0631 | 0.0005 | 0.0052 | 0.0040 |
| L4 | semi-automatic | 0.0007 | 0.0138 | 0.0173 | 0.0007 | 0.0044 | 0.0021 |
| L5 | manual | 0.0011 | 0.0105 | 0.0480 | 0.0006 | 0.0035 | 0.0019 |
| L6 | manual | 0.0007 | 0.0413 | 0.0444 | 0.0060 | 0.0058 | 0.0033 |
| L7 | manual | 0.0004 | 0.0144 | 0.0726 | 0.0005 | 0.0069 | 0.0040 |
| L8 | manual | 0.0004 | 0.0178 | 0.0566 | 0.0004 | 0.0039 | 0.0024 |
| L9 | manual | 0.0006 | 0.0101 | 0.0720 | 0.0004 | 0.0051 | 0.0043 |
| L10 | manual | 0.0000 | 0.0687 | 0.0310 | 0.0000 | 0.0000 | 0.0000 |
| L11 | manual | 0.0013 | 0.0210 | 0.0630 | 0.0005 | 0.0052 | 0.0040 |
| L12 | semi-automatic | 0.0009 | 0.0256 | 0.0266 | 0.0008 | 0.0045 | 0.0016 |
| L13 | semi-automatic | 0.0005 | 0.0030 | 0.0241 | 0.0014 | 0.0089 | 0.0038 |
|  | manual | 0.0005 | 0.0142 | 0.0759 | 0.0005 | 0.0044 | 0.0027 |
| L14 | manual | 0.0021 | 0.0470 | 0.0498 | 0.0017 | 0.0083 | 0.0026 |


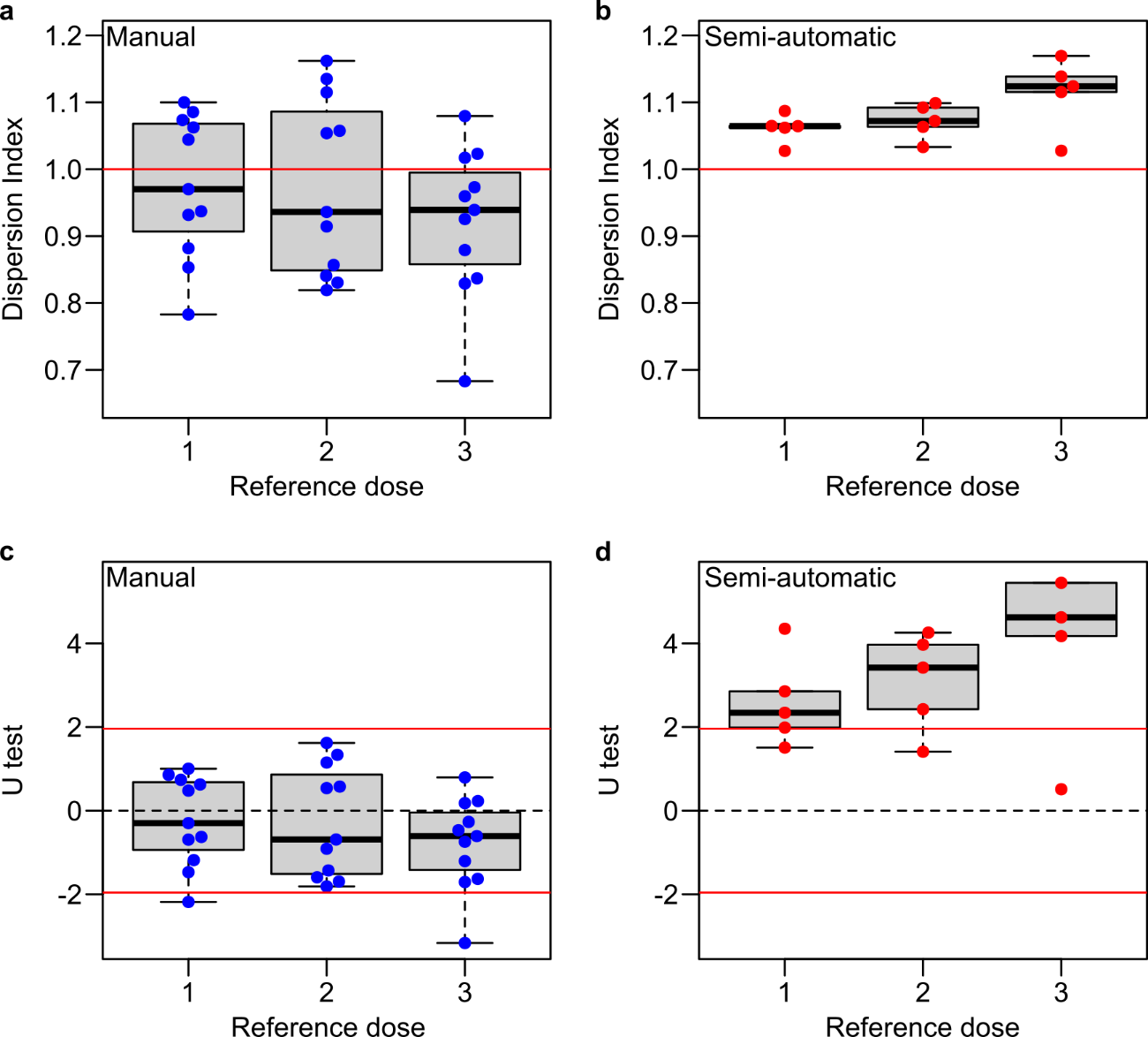


**Supplementary Figure S1:** Dispersion indices (**A&B**) and U test statistics are shown for manual (blue) and semi-automatic scoring. U>1.96 (red horizontal lines) is commonly used as an indication for a heterogeneous exposure.


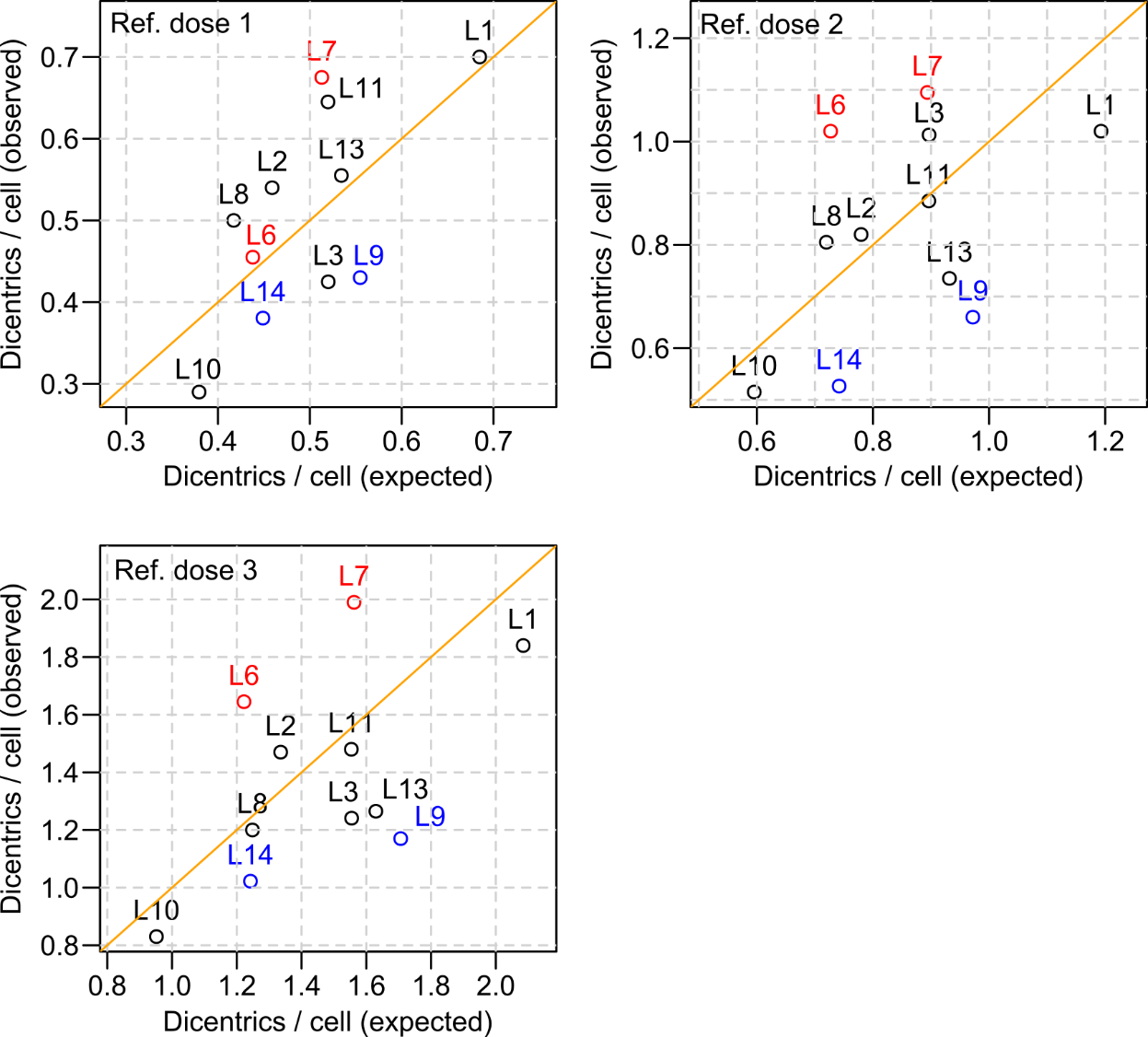


**Supplementary Figure S2:** Scatterplots for each reference dose, showing the association between the observed dicentric yield and the yield expected from the given reference dose and the calibration curve used by each participant for manual scoring. The expected yield was calculated by using the reference dose based on the dose definition (air kerma, dose to blood) matching the dose definition of the applied calibration curve (air kerma, dose to water). The orange line indicates the bisecting line and results close to the line indicate a good correspondence between the calibration curve and the results estimated by the participant. Laboratories where the results might indicate a systematic deviation between the expected and observed yields are labelled in red (overestimation) and blue (underestimation). The results of a participating laboratory were defined to show systematic under-/overestimation if 1) all three samples were consistently under-/overestimated and 2) if at least two of the 95% confidence intervals did not include the reference dose.


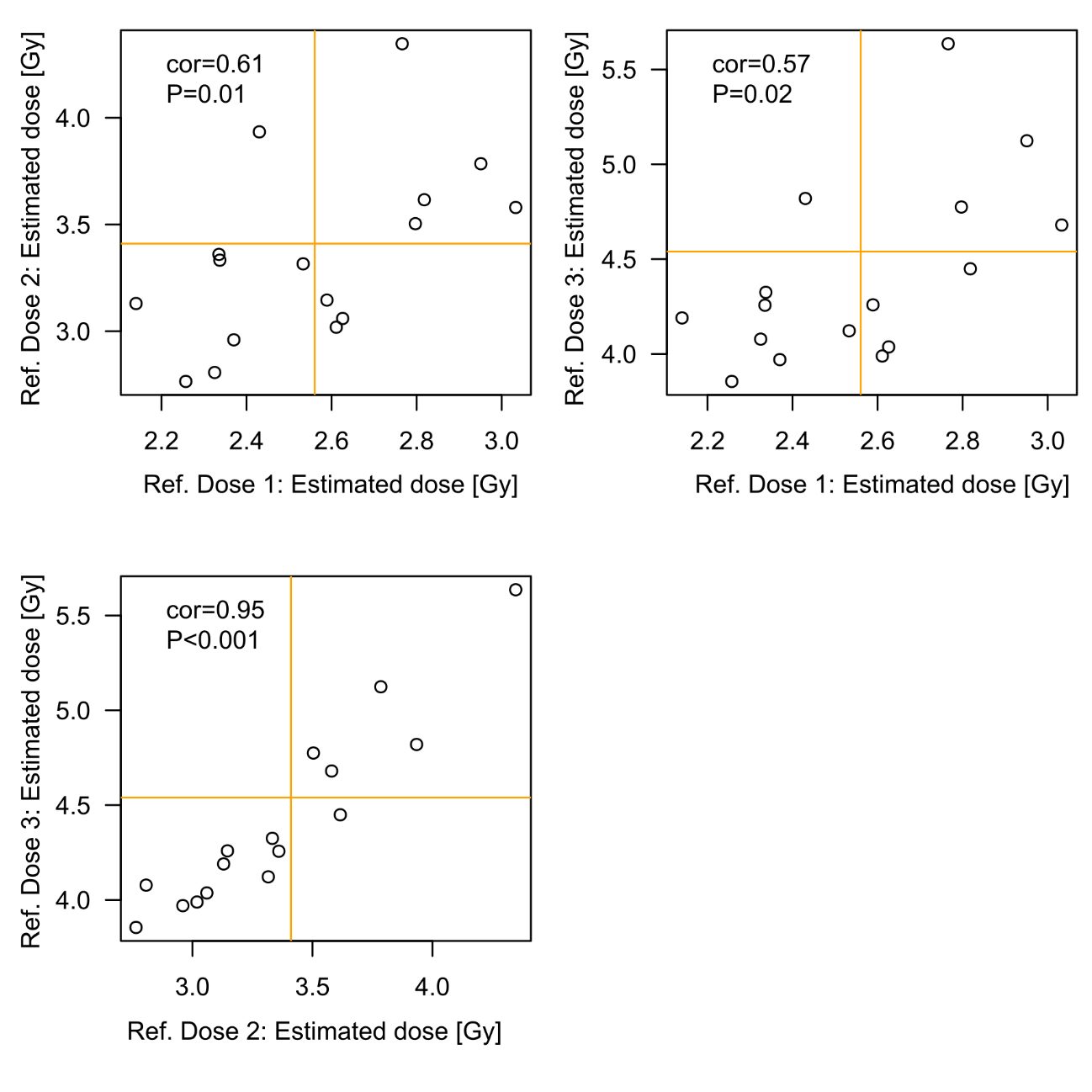


**Supplementary Figure S3:** Scatterplots showing the correlations (Spearman rank correlation) of the doses estimated by each participant between all pairs of blind samples. Significant correlations indicate that the same participants estimated high or low doses for both blind samples. Orange vertical and horizontal lines show the reference doses in terms of air kerma.
